# Identity and Seasonal Abundance of Beneficial Arthropods Associated with Big Sagebrush (*Artemisia tridentata*) in Central Washington State, USA

**DOI:** 10.3390/insects9030076

**Published:** 2018-06-28

**Authors:** David G. James, Lorraine Seymour, Gerry Lauby, Katie Buckley

**Affiliations:** Department of Entomology, Washington State University, Irrigated Agriculture Research and Extension Center, 24106 North Bunn Road, Prosser, WA 99350, USA; lorraine.seymour@pnnl.gov (L.S.); glauby@wsu.edu (G.L.); katie.buckley@wsu.edu (K.B.)

**Keywords:** predators, parasitoids, pollinators, crop pest management, sagebrush steppe, blooming, non-blooming

## Abstract

Big sagebrush (*Artemisia tridentata*) characterizes and dominates the sagebrush steppe, the largest temperate semi-desert ecosystem in North America. The beneficial arthropod fauna hosted by *A. tridentata* is poorly known but could be of importance to nearby agriculture seeking to exploit biologically-based pest management. Over four years, we identified and assessed the seasonal abundance of beneficial arthropods (predators, parasitoids, pollinators) associated with *A. tridentata* during spring to autumn in the Yakima Valley of central Washington using sticky traps. During 2011–2014, 207 sticky traps were placed on non-blooming and blooming *A. tridentata* plants for a total of 966 trapping days. Overall, across all seasons, we trapped 259.7 beneficial arthropods per trap and 92% of these were parasitoid wasps. Significantly greater numbers of beneficial arthropods were associated with blooming *A. tridentata* during autumn (410/trap) than non-blooming plants in the spring (181.3/trap) or summer (85.1/trap). Parasitoid wasps and predatory true bugs were most abundant during the autumn, but ladybeetles, lacewings, spiders, bees, and predatory thrips were most common during spring. The association of high numbers of predators, parasitoids, and pollinators with *A. tridentata* during blooming and non-blooming periods indicates that this plant is an important reservoir of beneficial arthropods in the sagebrush steppe of central Washington. Consequently, biologically-based pest management programs in central Washington may benefit from careful management and retention of *A. tridentata* plants on crop borders.

## 1. Introduction

Big sagebrush (*Artemisia tridentata* Nuttall) is a long-lived, widespread shrub that characterizes and dominates the shrub or sagebrush steppe, the largest temperate semi-desert ecosystem in North America [1,2]. It occurs from southern British Columbia to northern Baja Mexico and from the Dakotas and Nebraska to Washington, Oregon, and California, although its range has been greatly reduced and fragmented in recent decades [3]. Before agricultural development, the sagebrush steppe covered an estimated 38.6 million square kilometers, but today it occupies about half (56%) of its historic range [3]. The arthropod fauna of *A. tridentata* has been little studied, although recent research in Idaho indicates that the assemblage is large and diverse [4,5,6]. Specific arthropod species or communities and dynamics on *A. tridentata* have also been studied in Wyoming [7], California [8], Oregon [9], Utah [10,11], and Washington [12]. Numbers of arthropod species recorded on *A. tridentata* range from 106 to 232 in Idaho [4,6] and 168 in central Oregon [9]. A diverse and relatively large fauna (65 taxa) of beneficial insects and spiders was recorded on *A. tridentata* during spring to autumn in central Washington [12].

Agricultural development has occurred in many areas of the sagebrush steppe in the Pacific Northwest and is a significant driver in the decline of this ecosystem [3]. For example, large areas of sagebrush have been cleared in central Washington over the past 70 years to make way for extensive agricultural production based on wheat, potatoes, pome fruit, grapes, hops, and other horticultural and broadacre crops [13]. As crop pest management moves away from intensive chemical control to more biologically-based systems [14], increased attention is being given to the role and importance of natural plant communities in providing resources for beneficial arthropods [15]. Restoration of native plants and native plant communities can enhance populations of natural enemies of pest species, improving conservation biological control and reducing chemical inputs and costs in crop production [16]. Habitat restoration in and around croplands has also shown value for conserving wildlife including reptiles and mammals [17], birds [18], and butterflies [19].

Habitat restoration on cultivated land in central Washington agriculture occupying former sagebrush-steppe landscapes must consider the importance of *A. tridentata* as a host for beneficial arthropods, given its dominance in the sagebrush-steppe landscape. Previous studies have shown the importance of other sagebrush-steppe and riparian native plants like desert buckwheats (*Eriogonum* spp.), nettles (*Urtica* spp.), and milkweeds (*Asclepias* spp.) in attracting and retaining a range of beneficial arthropods [20,21,22]. While buckwheats occupy a similar area to *A. tridentata* (although plant densities are much smaller), nettles and milkweeds are generally confined to riparian zones within the sagebrush steppe. Seven native plants (including *A. tridentata*) surveyed in eastern Washington hosted varying numbers of predatory and parasitic arthropods [12].

This study identifies the major groups of beneficial arthropods (predators, parasitoids, pollinators) in the Yakima Valley of central Washington that are associated with *A. tridentata* and provides data on their seasonal abundance.

## 2. Materials and Methods

### 2.1. Sites

**Figure 1:**
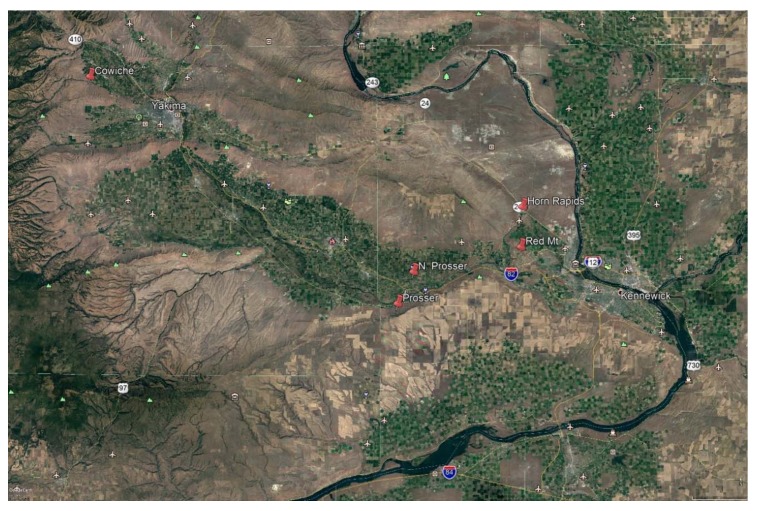
Eastern Washington State, USA, showing locations (red pins) of *Artemisia tridentata* sampled for beneficial arthropods.

This study was conducted over four seasons (2011–2014) in central Washington by counting and identifying beneficial arthropods associated with big sagebrush (*Artemisia tridentata* Nutt.) using transparent sticky traps. *Artemisia tridentata* plants were located in natural areas at five locations in the Yakima Valley (Red Mountain (46.17° N, 119.26° W), Cowiche Mountain (46.39° N, 120.46° W), Prosser (46.14° N, 119.42° W), North Prosser (46.18° N, 119.45° W), Horn Rapids (46.22° N, 119.26° W)) (Figure 1).

### 2.2. Traps and Trapping

Transparent sticky traps (WindowBugCatcher, 40.6 × 12.1 cm^2^, Alpha Scents Inc., Portland, OR, USA) were used, so that trap color did not influence insect attraction. At each site and on each occasion, a single trap was placed on each of three plants. Plants with traps were at least 5 m from other plants/traps and traps were attached to plants 1–2 m above ground level using flexible wires. Traps were left in place for 12–14 days before being removed and replaced. In less than 20 instances follow-up trapping occurred on the same plants (when plant numbers were limited) but usually different plants were chosen. Trapping was conducted during spring (April–May 2012, 2014: 224 total trapping days, non-blooming), summer (June–August 2012, 2014: 196 total trapping days non-blooming), and autumn (late September–October 2011, 2012, 2013: 546 total trapping days, blooming) using a total of 207 sticky traps. Availability of resources and funding dictated trapping regimes within and across years. During blooming, traps were placed over flowers. Traps collected from the field were transported to the laboratory and stored at −30 °C until examined under a stereomicroscope.

### 2.3. Arthropod Identification

All arthropods were identified to family or species and counted. The incidence and abundance of 34 species, genera, or groups of beneficial insects and spiders were recorded (Table 1). Numbers of leafhoppers (*Erythroneura* spp.) were also recorded.

Beneficial arthropods were condensed into 10 categories for analysis: lacewings (Chrysopidae), ladybeetles (Coccinellidae), predatory true bugs (Miridae, Anthocoridae), predatory thrips (Aeolothripidae), carnivorous (predatory and parasitoid) flies (Syrphidae, Empididae, Dolichopodidae, Tachinidae), ichneumonid and braconid wasps (Ichneumonidae, Braconidae), *Anagrus* wasps (Mymaridae), other parasitoid Hymenoptera (Pteromalidae, Eulophidae, Trichogrammatidae, Scelionidae), spiders (Linyphiidae, Oxyopidae, Clubionidae, Thomisidae, Salticidae), and bees (Apoidea). Bumblebees and larger wasps such as yellowjackets and hornets were usually able to extricate themselves from the sticky material and were rarely trapped and thus not recorded.

### 2.4. Data Analysis

Each site was considered a unit of replication and differences in numbers of arthropods trapped between seasons and years were tested using repeated-measures analysis of variance (ANOVA) with means separated using the Holm-Sidak method for comparing multiple groups (SigmaStat Version 3.0. SPSS Inc., Chicago, IL, USA). Trapping data were log (log x) transformed prior to analyses to improve normality of variances and then back-transformed for reporting.

## 3. Results

No differences in arthropods attracted in each season (spring, summer, autumn) were detected between years or sites and data obtained for each season during the study were combined for analysis. Beneficial arthropods dominated trap catches throughout the study. No herbivorous insects were captured in large numbers. Small numbers (<1 individual/trap, all years combined) of thrips (*Frankliniella* spp.), leafhoppers (*Empoasca* spp.), and Lygus bugs (*Lygus* spp.) were recorded but not presented. Grape leafhoppers (*Erythroneura* spp.) were the most common herbivores, especially in the autumn (mean ± SE: 28.7 ± 18.4 individuals/trap) and spring (11.0 ± 5.0 individuals/trap) (all years combined). 

**Figure 2:**
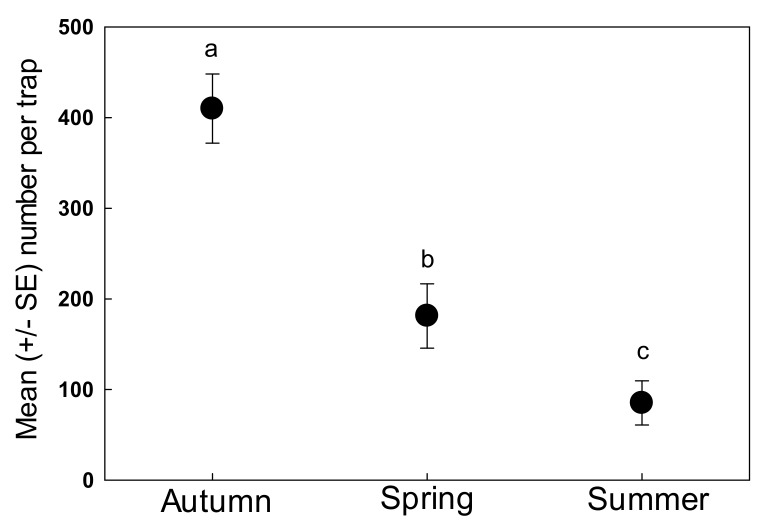
Seasonal abundance of all arthropods associated with *A. tridentata* in the Yakima Valley, Washington. Different letters denote significant differences (*p* < 0.001).

**Figure 3:**
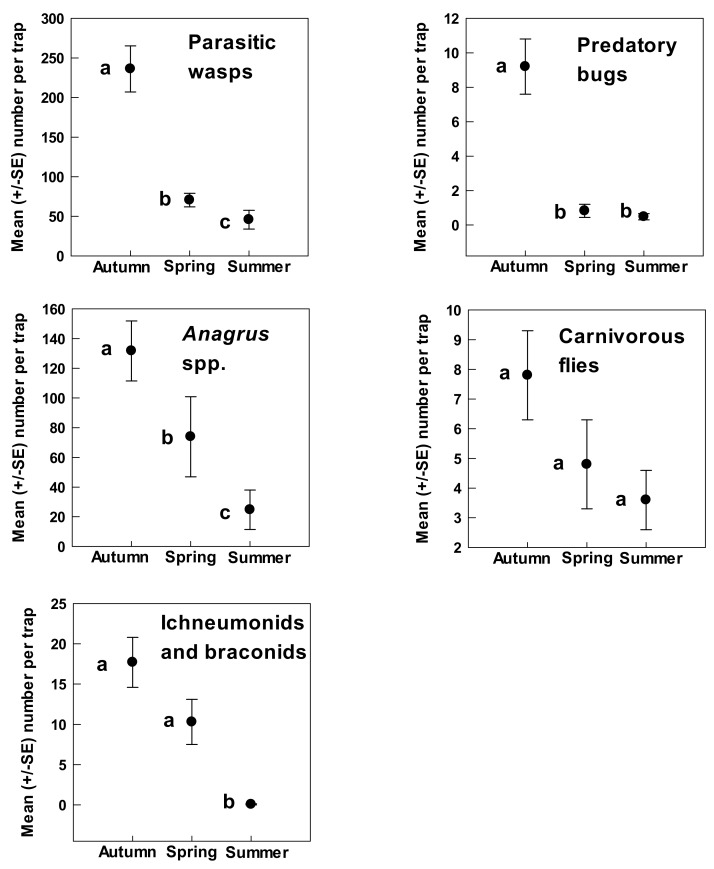
Beneficial insects associated with *A. tridentata* that showed greatest abundance during fall. Different letters denote significant differences (*p* < 0.001).

Combining all categories and analyzed over the entire seasonal study, the mean number of beneficial arthropod individuals attracted was 259.7 ± 68.8 individuals/trap. The majority (92%) of these were hymenopteran parasitoids (238.1 ± 65.8 individuals/trap). Significantly greater numbers of beneficial arthropods were associated with A. *tridentata* during autumn (410 ± 38.2 individuals/trap) than either spring (181.3 ± 35.6 individuals/trap) or summer (85.1 ± 24.4 individuals/trap) (*F* = 28.06, df 2, 66; *p* < 0.001; Figure 2). Numbers during spring were significantly greater than in the summer. Numbers of hymenopteran parasitoids were also higher during autumn than in spring or summer. Ichneumonid and braconid wasps were significantly more abundant in autumn than spring or summer and more abundant in spring than summer (*F* = 15.43, df 2, 67; *p* < 0.001; Figure 3). The mymarid wasps, *Anagrus* spp., and the remaining hymenopteran families followed the same trend (*Anagrus* spp. *F* = 31.25, df 2, 67; *p* < 0.001; Other Hymenoptera: *F* = 31.25, df 2, 67; *p* < 0.001; Figure 3). Other beneficial insect groups that were more abundant in the autumn than spring or summer included predatory true bugs (9.2/trap vs. 0.48–0.82/trap) (*F* = 23.19, df 2, 67; *p* < 0.001; Figure 3) and carnivorous flies (7.8 individuals/trap vs. 3.6–4.8 individuals/trap), although the latter were not significantly so (*F* = 1.55, df 2, 67; *p* = 0.22; Figure 3).

**Figure 4:**
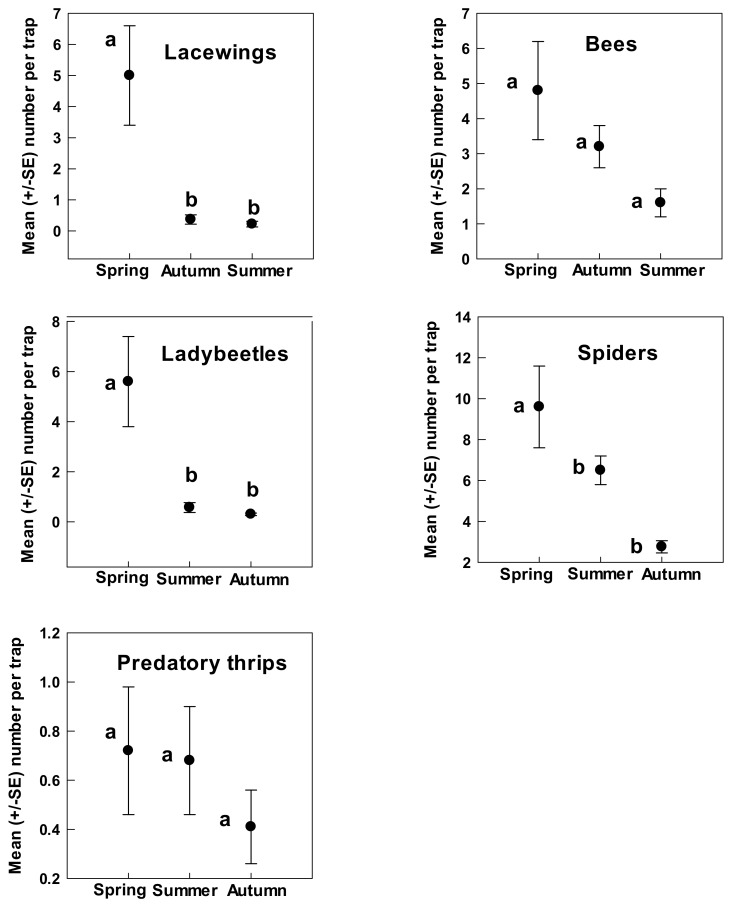
Beneficial arthropods associated with *A. tridentata* that showed greatest abundance during spring. Different letters denote significant differences (*p* < 0.001).

Three groups of arthropods were significantly more abundant on *A. tridentata* during spring than either summer or autumn. Ladybeetles and lacewings were common in spring (5.0–5.6 individuals/trap) but rarely seen during summer and autumn (0.22–0.57 individuals/trap) (ladybeetles: *F* = 22.97, df 2, 67; *p* < 0.001; lacewings: *F* = 31.25, df 2, 67; *p* < 0.001; Figure 4). Spiders were common in spring (9.6 individuals/trap) with numbers declining in summer (6.5 individuals/trap) to low numbers in fall (2.7 individuals/trap) (*F* = 13.35, df 2, 62; *p* < 0.001; Figure 4). Bees (4.8 individuals/trap) and predatory thrips (0.72 individuals/trap) were commonest in spring but not significantly greater than numbers trapped in spring and autumn (bees: *F* = 1.85, df 2, 67; *p* = 0.17; predatory thrips: *F* = 2.27, df 2, 67; *p* = 0.11; Figure 4).

## 4. Discussion

*Artemisia tridentata* dominates the sagebrush-steppe ecosystem throughout the Pacific Northwest and supports a diverse fauna of arthropods [4,5,12]. Our study provides evidence for the role of *A. tridentata* in central Washington as a reservoir of a large number of beneficial arthropods, primarily hymenopteran parasitoids throughout the growing season but particularly during autumn and spring. More than 90% of the beneficial arthropod individuals we recorded on *A. tridentata* were parasitoid wasps. A large proportion (36%) of these were individuals of *Anagrus* spp. (Mymaridae), important biological control agents of grape leafhoppers in wine and juice grape vineyards in central Washington [23]. *Anagrus* spp. responsible for biological control of grape leafhoppers in eastern Washington (*A. daanei*, *A. erythroneurae*, *A. tretiakovae*) [23] were among the species recorded from *A. tridentata* in the Yakima Valley. Predatory true bugs, primarily anthocorids (*Orius* spp.) and geocorids (*Geocoris* spp.), were the most numerous predatory insects associated with *A. tridentata*. Increased abundance of parasitoid Hymenoptera, predatory true bugs, and carnivorous flies during the autumn, when *A. tridentata* blooms, resulted in the greatest seasonal density of beneficial arthropods. However, five groups of beneficial arthropods (lacewings, ladybeetles, predatory thrips, spiders, and bees) showed greatest abundance during spring when no blooms were present. Curiously, we did not trap large numbers of herbivorous insects. Large populations of herbivorous insects like aphids and grasshoppers had a major impact on growth, flower production, and seed set on *A. tridentata* in Utah [5]. Few grasshoppers were seen at our sampling sites and aphid infestations were not noted. The only abundant herbivores were grape leafhoppers, primarily in autumn and spring, and these probably used *A. tridentata* for overwintering rather than as a food source [23].

*Artemisia tridentata* is the dominant plant of the sagebrush-steppe ecosystem and might be expected to play an important role in the ecology of many arthropod species living within this system. Our study suggests that its significance extends beyond the provision of nectar during blooming because high numbers of beneficial arthropods were also present during non-blooming periods, particularly in the spring. Beneficial arthropods are associated with *A. tridentata* pre- and post-flowering in eastern Washington, but numbers are about 25–30% of those recorded during flowering [12]. During a broader study of beneficial arthropods trapped on ~100 Yakima Valley native plant species during 2010–2014 (James, unpublished), no other species matched or exceeded the mean number of individuals trapped on *A. tridentata* (259.7 individuals/trap). For example, the mean number of all beneficial arthropods trapped on buckwheats (*Eriogonum* spp.) ranges from 48.5 to 167.7 individuals/trap [20], on milkweeds (*Asclepias* spp.) 126–128 individuals/trap [22], and on stinging nettle (*Urtica dioica* L.) 140 individuals/trap [21]. In many instances, the numbers of beneficial arthropods attracted to non-flowering *A. tridentata* exceeded numbers trapped on flowering native plant species. The association of beneficial arthropods with non-flowering plants has been little studied, but our results and those of [21,24,25] indicate that some plants are attractive to predators and parasitoids throughout the growing season. Some predators and parasitoids may respond to plant cues other than chemicals associated with flowers and may be seeking plant-provided benefits like refuge, mating sites, hosts, and alternative food sources. The relatively large stature and dense structure of *A. tridentata* plants likely makes them an important refuge for arthropods, protecting them from natural enemies and buffering against extremes of heat and cold.

The extremely large numbers of *Anagrus* spp. associated with *A. tridentata* in autumn (132 individuals/trap) followed by relatively large numbers in spring (74 individuals/trap) suggests that this species may use the plant for overwintering. However, *Anagrus* spp. overwinter as eggs within leafhopper eggs. We did not examine twigs and foliage of *A. tridentata* for host leafhoppers, but small numbers of *Empoasca* spp. leafhoppers were occasionally trapped during the study. A number of leafhopper species have been reported on *A. tridentata* in Utah [6] and Oregon [9] and it is likely that leafhoppers also feed on sagebrush in Washington. The relationship between *Anagrus* spp. and *A*. *tridentata* deserves further investigation, especially given the importance of these parasitoids to biological control of grape leafhoppers in Washington vineyards [23].

Agricultural development in central Washington invariably involves the removal and fragmentation of shrub-steppe habitat [13]. Sagebrush is the primary plant removed and our study suggests that this also removes a valuable resource of beneficial arthropods that could be utilized in crop pest management. Many of the beneficial arthropods trapped in this study, for example, generalist predators in the families Anthocoridae, Geocoridae, Chrysopidae, and Coccinellidae, are important agents of conservation biological control in regional horticultural crops [26,27]. The degree to which beneficial arthropods move from *A. tridentata* into cropping ecosystems remains unknown and needs further research. However, there are many documented examples of beneficial insect movement from natural ecosystems into adjacent cropland [28,29], and it is likely that at least some movement occurs from *A. tridentata* to nearby crops. In many instances, agricultural land clearing removes far more *A. tridentata* than is actually necessary for crop establishment. Clearing may extend for many meters beyond the crop zone to create a ‘neutral space’ for reasons of ‘tidiness’ or creating a perceived pest-free zone. Big sagebrush is not known to harbor significant populations of important crop pests in eastern Washington and our study, with few herbivorous arthropods recorded, supports this notion. We suggest that the clearing of *A. tridentata* during the creation of new agricultural sites be limited as far as possible to retain the important ecosystem services provided by this plant. Fragmented shrub-steppe lands in central Washington adjacent to cropland support a high diversity of beneficial arthropods, although populations were smaller than in undisturbed areas [30]. Thus, it is likely that even limited areas and numbers of *A. tridentata* plants will serve as reservoirs of beneficial arthropods and aid in natural pest management in adjacent cropland.

## 5. Conclusions

This study provides compelling evidence for the functioning of big sagebrush, *A. tridentata*, in central Washington as a reservoir of beneficial arthropods throughout spring, summer, and autumn. The majority of beneficial arthropods associated with *A. tridentata* in this study were hymenopteran parasitoids and greatest numbers occurred during blooming in autumn. Ladybeetles, carnivorous flies, and spiders were commonest during spring. The association of high numbers of predators, parasitoids, and pollinators with *A. tridentata* during blooming and non-blooming periods indicates the potential importance of this wide-ranging landscape plant in the Pacific Northwest to arthropods within the shrub-steppe ecosystem. Ecosystem services like pollination and biological control in agriculture may benefit from proximity to expanses of *A. tridentata* and we recommend utilization of this natural plant resource in planning and maintenance of agricultural lands.

## Figures and Tables

**Table 1:** Categories of beneficial arthropods identified and recorded in this study, along with species, genera, and families within each category.

Beneficial Insect Categories	Species, Genera, or Family Included
Neuroptera (Lacewings)	*Chrysoperla plorabunda* (Fitch) *Chrysopa nigricornis* Burmeister *Chrysopa coloradensis* Banks *Chrysopa oculata* Say
Coccinellidae (Ladybeetles)	*Harmonia axyridis* (Pallas) *Coccinella septempunctata* L. *Coccinella transversogutatta* Mulsant *Hippodamia convergens* (Guerin-Meneville) *Stethorus picipes* Casey *Stethorus punctillum* (Weise)
Heteroptera (Predatory true bugs)	*Deraeocoris brevis* (Uhler) *Geocoris pallens* Stal *Orius* spp.
Aeolothripidae (Predatory thrips)	*Franklinothrips* spp. *Aeolothrips* spp.
Diptera (Predatory and parasitic flies)	Empididae Syrphidae Dolichopodidae Tachinidae
Hymenoptera: Ichneumonidae and Braconidae (Ichneumonid and braconid wasps)	Ichneumonidae Braconidae
Mymaridae (Fairy wasps)	*Anagrus* spp.
Other Hymenoptera	Pteromalidae, Eulophidae, Trichogrammatidae, Scelionidae
Araneae (Spiders)	Linyphiidae, Oxyopidae, Clubionidae, Thomisidae, Salticidae
Apoidea (Bees)	*Apis mellifera* L., Andrenidae, Halictidae, Megachilidae, Apidae, Colletidae
